# Transforming Perspectives in Cardiac Cell Therapy: Hypothesis and Commentary Following Updated Results of a Pilot Study Investigating Very Long-Term Clinical Outcomes in Severe AMI Patients Following Trans-Epicardial Injection of Peripheral Blood CD34^+^ Cells

**DOI:** 10.1007/s12015-023-10643-w

**Published:** 2023-10-20

**Authors:** Philippe Hénon, Nicolas Bischoff, Robert Dallemand

**Affiliations:** 1 Institut de Recherche en Hématologie Et Transplantation, Hôpital du Hasenrain, 87 Avenue d’Altkirch, 68100 Mulhouse, France; 2 CellProthera SAS, 12 Rue du Parc, 68100 Mulhouse, France; 3 Département de Chirurgie Cardio-Thoracique, Groupe Hospitalier Régional Mulhouse Sud-Alsace, 20 Rue du Docteur Laënnec, 68100 Mulhouse, France

**Keywords:** Myocardial infarction, CABG, Cell therapy, CD34 + cells, Long-term outcome

## Abstract

Ischemic heart attack is the leading cause of death worldwide. Ten percent of cases will die within an hour. Of the survivors, around 30% will have suffered a severe infarction which will lead to the irreparable destruction of 1 to 2 billion myocardial cells, causing an irreversible secondary heart failure with a poor prognosis in the short. The heart is a totally differentiated organ with a very low capacity for self-regeneration. No current treatment can prevent this fatal outcome, but only slow it down. For these reasons, cell therapy has generated enormous hope, but achieved somewhat disappointing results, depending on the type/source of cells which were used. From the end of 2002, our group conducted a Pilot study using immuno-selected autologous peripheral-blood (PB) CD34^+^ cells in a small cohort of patients who had experienced a heart attack with poor prognosis. Three of these patients were immediately considered for heart transplant but lacked a readily available donor. CD34^+^ cells were trans-epicardially delivered at the end of a coronary artery by-pass graft (CABG) operation without reperfusing the ischemic area, which was performed on a compassionate basis. All but one patient showed a marked and sustained improvement in their cardiac function parameters from the baseline values, associated with both cardiac tissue repair and revascularization, as demonstrated by PetScan examination. The patients’ outcomes have been recently updated. Six out of seven patients have survived in good enough conditions for at least 12 years after cell therapy, including those three initially recommended for heart transplant and who have avoided it. Presently, five out of seven patients are still alive with an average follow-up of 17 years (range 16–20 years), which is very unusual after CABG for patients with such a poor initially prognosis.

**Graphical Abstract:**

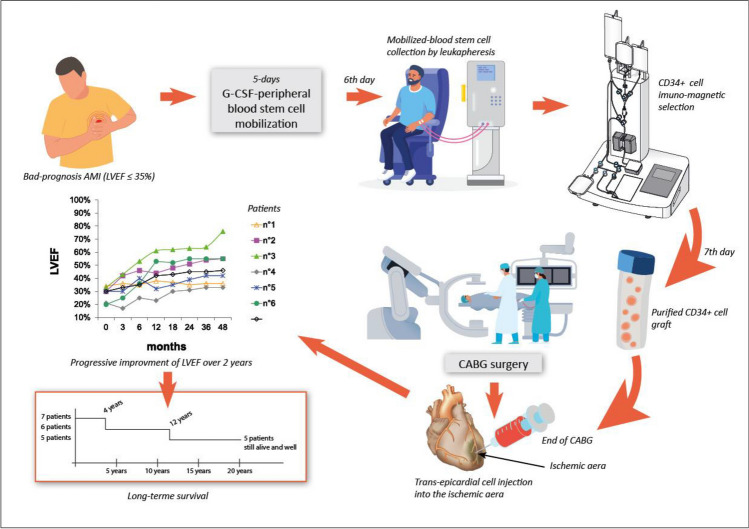

## Introduction

About 3.5 million cases of heart attack occur each year in the seven most populous European countries, the USA and Japan (i.e., one/seventh of the world population). Presently, about 10% of cases will die within an hour. Of the survivors, around 30% will have suffered a severe infarction which will inevitably lead them to chronic heart failure (CHF) with a poor prognosis in the short term. According to the results of the Framingham study over a period of 15 years, 15% did not survive the year and, at 5 years the survival rate was only 25% in men and 38% in women [1]. Statistics on 22,000 people suffering from acute myocardial infarctions (AMI), recently reported by the Swedish National Board of Health and Welfare, covering the 2006–2020 period, showed that 33% of the patients (men as well as women) still died within a year [2]. Even if short-term mortality seems to have decreased within the past decade, the 10-year mortality evaluated in almost 4 million patients who survived the acute period of AMI was still 72.7% [3]. Ischemic heart attack remains the leading cause of death worldwide and is responsible for about 16% of total deaths according to a World Health Organization (WHO) estimate [4].

A severe AMI will lead to the irreparable destruction of 1 to 2 billion myocardial cells (cardiomyocytes), and the heart is indeed a totally differentiated organ with a very low capacity for self-regeneration [5]. In the weeks following an infarction, the intact cardiomyocytes surrounding the ischemic zone will undergo hypertrophy and, in association with the reactive inflammatory phenomena, will generate the remodeling of this area while fibrosis is established which will "fill the void" left by the destroyed cells. This set of processes results in a more or less extensive non-contractile part of the heart, leading to progressively increasing secondary heart failure. No current treatment can prevent this evolution, but only slow it down, without preventing the eventual fatal outcome.

For these reasons, cell therapy has generated enormous hope within the past two decades, but often achieved disappointing results. CD34^+^ stem cells are among the various types of cells used in cardiac indications. We ourselves carried out a pilot clinical trial in the early 2000s in which a small series of patients with recent severe AMI—whose statistical life expectancy was less than 2 years, particularly for three of them who were scheduled for heart transplantation [6]—received an intracardiac injection of purified autologous peripheral blood (PB)- CD34^+^ cells into the ischemic cardiac injury at the end of a CABG. This therapeutic technique partially regenerated the heart tissue and increased cardiac function in all patients, except one, and prevented the onset of secondary CHF. The results of the patients’ assessments and their outcomes at 2 years after the transplant have already been summarized in [6]. However further detailed outcomes of each patient have been recently updated and are reported here.

## Patients and Methods

From December, 2002, to January, 2007, seven patients (5 males, 2 females, aged 33–72 years) who had experienced a myocardial infarction (MI) with a very poor prognosis were enrolled in a pilot clinical study to receive intra-epicardially autologous PB-CD34^+^ cells at the end of a CABG surgery, without reperfusing the ischemic area, performed on a compassionate basis. The main criteria for selecting the patients were: left ventricular (LV) transmural MI; age ≤ 75 years; left ventricle ejection fraction (LVEF) ≤ 35%; distinct area of akinesia corresponding to the infarct’s localization detected by bi-dimensional echocardiography (echo 2D at diagnosis -and further confirmed by tri-dimensional echocardiography (echo3D); distinct area of non-viable and non-perfused LV myocardium detected by a positron emission tomographic scan (PetScan) examination (PSE) after intravenous injections of ^18^Fi-FDG and of ^201^Ti-chloride, respectively; Class 4 exercise capacity according to the criteria of the New York Heart Association (NYHA); a non-reperfused ischemic area; and an indication for CABG surgery on the coronary arteries other than the obstructed vessel(s).

After the patients signed the informed consent form, they were assessed with echo3D allowing the determination of LVEF, ^201^thalium scintigraphy, and PSE to ensure that they met the inclusion and none of the exclusion criteria, so that they could be enrolled. Once the date of CABG was scheduled, CD34^+^ cells were mobilized from the bone marrow (BM) into the PB by the sub-cutaneous administration of granulocyte-colony stimulating factor (G-CSF), at 10 µg/kg daily for five days. On the sixth day, leukapheresis (LKP) was performed with the goal of collecting at least 10^9^ cells, as recommended for further satisfactory selection procedure. When the collection was poor, a second LKP was performed early in the morning on the seventh day. The whole LKP products were then processed for CD34^+^ immunoselection with the clinical Isolex 300i magnetic cell-separation device (Baxter Healthcare, Deerfield, IL, USA). The cells were resuspended in 100 mL of human albumin and concentrated by mild centrifugation at a final graft volume of 15 mL.

CABG began as soon as the cell graft was available, and was carried out with a beating heart. The cell suspension was always infused throughout the LV wall’s ischemic area by several longitudinal and parallel injections just before the completion of the operation. The septum was never injected. The CABG and cell injection were performed 11 weeks after the AMI on average (range: 6—24 weeks) excepted in Patient#1 (8 years later).

Patients were further assessed by echo 3D at 3, 6, 12, 18, and 24 months, with ^201^Thalium scintigraphy and PSE at 6 and 12 months post-surgery.

## Follow-up and Long-Term Survey of the Patients

### Patient 1

Was male, 39 years-old, and a smoker. He had undergone an anterior MI by ostial obstruction of the left anterior descending (LAD) coronary artery (CA) in 1994. He began to suffer from refractory angina from the end of 2001. A coronarography performed in January 2002 showed, beside the ostial obstruction of the LAD, disseminated points of stenosis of the circumflex artery (Cx) and a full occlusion of the first marginal artery (Mg). A marked LV dilatation (220 mL) was associated with extensive anterior akinesia, apical dyskinesia and a calcified apical thrombus. ^201^Thallium scintigraphy both confirmed the scar of a former massive antero-lateral transmural MI and detected an area of antero-septal and apical non-viability, related to a more recent transmural MI. The LVEF was 42%.

The angina and the prognosis of the patient progressively worsened and a CABG combined with CD34^+^ cell injection was considered at the end of 2002. His LVEF was 35%, and PSE showed an extensive antero-septal and apical non-viability. His NYHA Class was 4. In total, 29.1 × 10^6^ CD34^+^ cells were trans-epicardially delivered at the end of a mono-CABG (Cx*)*. However, the MI area visually appeared to be totally sclerotic and calcified, which was not compatible with a satisfactory cell survival, thus making the potential success of the cell therapy doubtful. No adverse event (AE) related to injection of the cells, particularly arrhythmia, was observed.

Within the following weeks, his angina symptoms disappeared rapidly and the patient felt better as a result of the CABG, and his NYHA Class decreased from 4 to 3. However, assessments at 3, 6, and 12 months did not shown any improvement in the akinetic and/or dyskinetic areas, the cardiac tissue’s viability, LV volumes, and LVEF remained between 35 and 38%. For the following 3 years, the patient’s state remained globally stable. However, from 46 months post-CABG an acute cardiovascular decompensation occurred and the patient died at 48 months post-CABG.

### Patient 2

This patient was female, 49 years-old, overweight and a smoker, with high blood pressure, familial dyslipidemia, and previous left retinal artery thrombosis, On 3 December, 2002, she complained of « gastralgias» that increased on the next day, with the occurrence of intense dyspnea, sweating, restlessness, coughing and foamy sputum, which are symptoms of acute pulmonary oedema (APO). She was transferred to the emergency hospital 48 h after the appearance of the first symptoms. Treatment of the APO was immediately launched. A coronarography showed a tri-truncal obstruction of LAD2, Mg and diagonal-1(D1) CA, which were responsible for an extensive antero-apical as well as a transmural MI with severe alteration in LV function (LVEF 30%), and a truncal obstruction of the right CA. Extended antero-apical akinesia and a marked LV dilatation were recorded. The apex and one-half of the LV anterior wall did not fix ^18^FDG. The patient suffered from angina during effort and was classified as NYHA class 4.

The patient was immediately considered for heart transplantation, but lacked a readily available donor. She thus accepted to enter the protocol. In total, 40.3 × 10^6^ CD34^+^ cells were delivered at the end of a mono-CABG (LIMA-D1) performed on 14 January, 2003. However, 3 weeks later, she suddenly felt unwell with vomiting and spiking blood pressure. A tamponade was diagnosed and evacuated in an emergency procedure. An echo3D carried 2 days afterwards did not shown any sequalae of the tamponade when, interestingly, the LVEF had increased up to 46% associated with a significant improvement in the anterior wall kinetics and the LV’s volumes, while her NYHA Class decreased from 4 to 3.

**Fig. 1:**
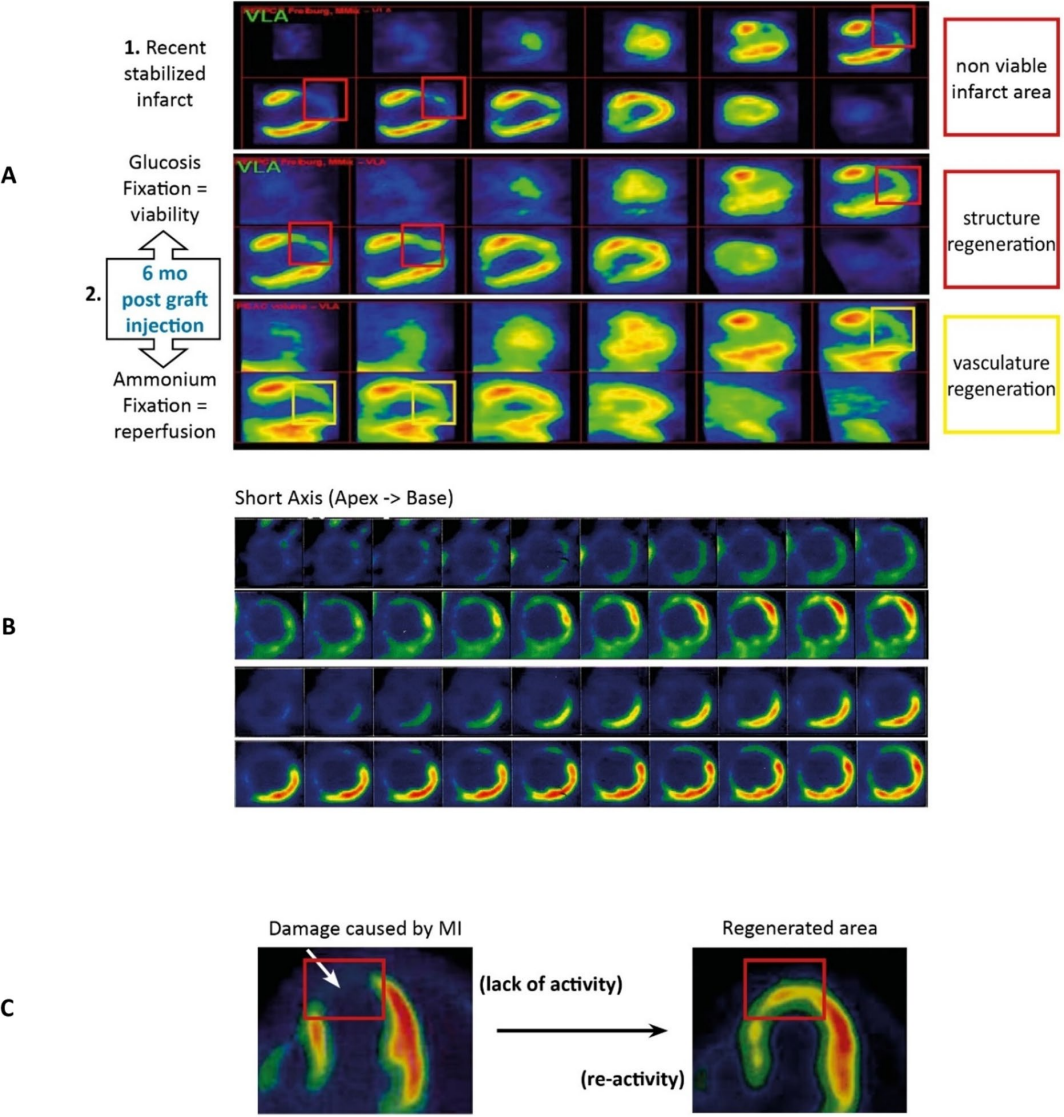
PetScan examinations of Patients 2, 4 and 5. **A**: Petscan of Patient 2. Upper raw: large non viability area (red square) in the left ventricle anterior wall before cells injection. Middle raw: cardiac tissue regeneration 6 months after cells injection, objectified by fixation of ^18^Fi-FDG into what was the dead area (red square). Lower raw: fixation of ^201^Ti-chloride 6 months after cells injection, showing the revascularization of the initially dead area (yellow square). **B**: Patient 4 ^18^Fi-FDG PetScan. Upper raw, before cells injection: non-viability of the myocardium involving the apex, almost all the anterior wall, the septum and the posterior wall from the apex to the middle of the ventricle. Lower raw, 1 year after cells injection: improvement of the viability in the basal parts of the anterior wall and of the septum, and in all dorsal wall. **C**: ^18^Fi-FDG PetScan images of Patient 5. Left: After AMI and before cells injection, the infarcted zone is inactive. Right: the same zone is again viable 1 year after cells injection

Her LV function still progressively improved, with her LVEF between 52 and 55%, a decrease of 10 mL in the LV end systolic volume (LVESV). Her LV’s anterior wall kinetics and fixation of ^18^FDG and of ^201^Ti-chloride at 6 months was almost normal, except in the apex area (Fig. 1A). The 12 months assessment confirmed both cardiac tissue repair/ revascularization and the resumption of subnormal kinetics of the LV anterior wall, while the apex remained mute and dyskinetic. NYHA Class still improved further from 3 to 1.

Throughout the following 12 years, the patient felt very well, living and working normally with her LVEF remaining stable around 55% On October 2015, a peripheral angioplasty was performed for an obstruction of the common femoral artery. In 2019, she underwent a stroke episode with hemiparesis and mild aphasia, from which she recovered well. Twenty years after cell therapy, she is still alive and well with stable LV function, and has avoided heart transplantation. She is now 69 years-old.

### Patient 3

This patient was female, 61 years-old and overweight, with diabetes mellitus, high blood pressure, and a prior transitory ischemic accident. On December 2002, the patient felt a transient thoracic pain associated with shortness of breath for a few minutes. As things seemed to recover well, the patient did not see a doctor for weeks. However, from the end of January 2003, she felt more and more tired and out of breath and was finally taken to the hospital 2 months after the occurrence of pauci-symptoms. The ECG showed the sequelae of anterior necrosis, and an anterior transmural MI of the LV wall, the antero-septal junction and the apical areas was diagnosed corresponding to a severe hypokinesia, related to a 90% stenosis of the proximal LAD2 at the junction with D1, and an obstruction of proximal D1.

At inclusion, her LVEF was 35%. ^18^FDG PSE showed the non-viability of a scar including the apex and the inferior third of the antero- lateral LV wall. The NYHA Class was 4. In total, 43.8 × 10^6^ CD34 + cells were delivered at the end of a mono-aorto-coronary CABG (LIMA-LVA) on 26 March, 2003. The patient was discharged from cardiac rehabilitation on 3 May, 2003. At this time, her LVEF had increased up to 45% while the hypokinesia slightly improved in the antero-lateral wall but not in the antero-septal junction or apex.

At 6-month follow-up (FU), improvement in her LV function was still more significant: LVEF was 53%, associated to an improvement in the antero-lateral wall contractility while the other ischemic areas remained hypokinetic, and a partial restoration of the viability of the antero-lateral wall was observed. The patient was well, living almost normally.

At 1-year FU, her LVEF was 63%. Only a slight hypokinesia persisted in the antero-septal junction and the intra-ventricular septum, associated with almost normal contractility and good cardiac tissue viability and perfusion of the LV antero-lateral wall. At 18 months post-operation, only the apex remained non-viable, her LVEF was 69%, and her NYHA Class was 1.

The patient did very well and was asymptomatic for 11 years, LVEF being stable between 65 and 70%. She was never hospitalized during this period. However, in 2014, the occurrence of an atrioventricular bundle branch block required the implantation of a pacemaker, In 2017 she underwent a second AMI, occurring from a tight stenosis of the common coronary artery, which was treated by angioplasty, and she recovered well. Presently, 20 years after cell therapy, she is in good condition for her age (80 years-old) with no symptom of heart failure (HF). The apex remains non-contractile and non-viable, but her LVEF is now stable between 40 and 45%.

### Patient 4

This patient was male, 33 years-old, alcoholic and a smoker. He had already suffered for several years from transient pre-cordial pain after vigorous exertion. On 7 March, 2006, after a prolonged effort removing snow, a brutal and intense squeezing thoracic pain suddenly occurred, irradiating to the left arm and the neck, associated with dyspnea, palpitations, tachycardia and vomiting. The patient was rapidly taken by the emergency service where a cardiogenic shock was performed. ECG and echo3D showed respectively an elevated ST and severe LV dilatation, a severe diffuse hypokinesia only sparing the basal third of the LV; his LVEF was 25%. A coronarography highlighted a bi-truncal coronary lesion with an old obstruction of the proximal LAD ostium and a sub-occlusive stenosis of the distal section of the middle right coronary artery. ^201^Thalllium scintigraphy confirmed the large transmural MI including an aneurismal apex, the antero-septal junction, two-thirds of the LV anterior wall, the intra-ventricular septum and the apical third of the postero-inferior wall. Because of the initial cardiogenic shock and the occurrence of several further episodes of ventricular tachycardia, an electric defibrillator was implanted on April 3^rd^.

Considering the young age of the patient and the very poor short-term prognosis, he was recommended for urgent heart transplantation, but this was refused by the transplant surgeons because of the medical context (alcoholism and smoking). Instead, the patient was enrolled in our study. His LVEF, LVESV and LVEDV were respectively 21%, 175 mL, and 240 mL, with a non-viable area corresponding to the infarct’s localization. The patient was very dyspneic and was not able to walk more than 10 m (NYHA Class 4).

In total, 107.6 × 10^6^ CD34^+^ cells were trans-epicardially delivered at the end of a mono-CABG (LAD) performed on 12 April, 2006. No AE was reported within the post-operative period and the patient progressively improved his physical capacities, with a 6 mn walking test of 400 m, and less dyspnea. He returned home on 13 May. Because of the severity of HF, he was followed every two weeks in outpatient consultation until the third month. At this time, he appeared less dyspneic (NYHA Class 2) and in better clinical state, but his cardiac parameters were significantly worsening: LVEF 15%; LVESV: 237 mL; LVEDV: 301 mL, and he showed no improvement in the kinetics. The patient was again recommended for heart transplantation, and accepted this time, but lacked an available donor. He continued to receive a symptomatic treatment only.

However, at the 6th-month assessment, not only was the patient still alive, but all evaluation parameters began to clearly improve: LVEF 22% and slight recovery of the viability and reperfusion of the postero-inferior wall. This improvement increased for months. The 1-year assessment showed good recovery of the viability and perfusion of the postero-inferior wall, and a partial recovery of the viability of the antero-lateral wall (Fig. 1B), corresponding to the improved kinetics of these areas and the LVEF (25%). At the 2-year follow-up (FU), his LVEF had gained 16 points (31%) from month 3, the patient was in good clinical condition without any symptoms of HF and had progressively resumed physical activities (NYHA Class 1). At 4 years, his LVEF remained stable at around 30%, the patient felt very well and was capable to ride a bike for 60 kms three times a week, to swim 100 m continuously, and to play basketball for half an hour. He walked 6 kms every day.

Things remained stable up to 2011, when he made a suicide attempt by drugs in the context of marital breakdown, which required hospitalization for 3 weeks in a psychiatric ward, but also had a toxic impact on his cardiac function: his LVEF felt to 25% within the subsequent weeks and continued to progressively decrease over the following months (12% one year after the suicide attempt) associated with an increase in the LV’s volumes, when NYHA Class 2 exertional dyspnea reappeared. Things stayed that way for the following years but the patient remained depressed and he made a second suicide attempt by ingesting car antifreeze, causing acute kidney failure which required several hemodialysis sessions, leading to a gradual recovery of kidney function.

Thankfully, the patient survived and is still alive, 17 years after cell therapy. He is now 50 years-old. The last cardiac assessment performed in January 2023, showed a clinical stability with a persisting NYHA Class 2 dyspnea, LVEF at 15%, and marked LV dilatation. However, he is not doing poorly and has avoided heart transplantation up to now.

### Patient 5

This patient was male, 72 years-old. He had experienced a previous inferior AMI by Cx obstruction in 1996, which was treated by angioplasty. In January 2006, the patient was hospitalized because of dyspnea and left latero-thoracic pain that had been progressively increasing for the last two months and was associated with transient atrial fibrillation episodes. An echo3D revealed a severe trans-mural MI, with akinesia of the postero-inferior wall and of the septum, related to the prior AMI, alongside severe antero-lateral hypokinesia, LV dilatation, and LVEF at 27%. A coronarography identified a bi-truncal involvement with a 70% stenosis of the LAD/ Dg2 crossroad and a sub-occlusive stenosis of the proximal Cx, which was immediately stented. Two months later, the median LAD was also stented. The patient was well until the end of June when he felt dyspneic again (grade 3) with severe angina resulting from the slightest effort. His LVEF was 30%; the apex, the antero-apical wall and the basal septum were akinetic, as well as the postero-inferior wall, the site of the first AMI. A new coronarography showed restenosis of both the LAD and Cx at the stent’s level. A PSE showed two separate areas of non-viability: one in the postero-inferior wall, corresponding to the scar of the first AMI, the second was larger, comprising the apex, the third inferior segment of the anterior wall and the apical part of the septum. The patient was then scheduled for CABG plus CD34^+^ cell therapy, and recommended for further heart transplantation.

In total, 41 × 10^6^ CD34^+^ cells were intra-epicardially delivered at the end of a triple CABG (LAD, Dg, Cx) performed on 10 August, 2006.The post-operative period was good enough, the patient became asymptomatic and recovered some physical capacity, improving still more within the following months. At the 6-month FU, his LVEF reached 40%, correlated with a recovery of the antero-septo-lateral area’s viability and kinetics. However, a supraventricular arrhythmia access occurred in the ninth month, resulting in a sudden decompensation of the left heart function, dyspnea (NYHA Class 2) and a drastic drop in LVEF (20%), requiring the implantation of a defibrillator. He then improved with the LVEF value progressively increasing once more. Furthermore, at the 1-year FU, a significant regeneration and reperfusion of the ischemic zone that had been treated by cell injection was recorded, contrasting with the « mute» scar of the oldest infero-lateral AMI, which thus appeared as an internal negative control (Fig. 1C).

Throughout the following 10 years, the patient did very well, leading a normal life with good enough physical capacity (walking distance: 6kms), while his LVEF remained stable around 40–45%; he was classified NYHA Class 1, and he no longer required heart transplantation. However on 15 March, 2018, almost 12 years after CD34 + cell therapy, he suddenly died of a stroke during the night. He was 84 years-old.

### Patient 6

This patient was male, 63 years-old, and an ex-smoker with diabetes mellitus. An antero-septal MI was fortuitously discovered during a pre-coloscopy assessment at the beginning of October, 2006, but had probably occurred in July. His LVEF was 25% associated with mild LV dilatation and an apical aneurysm.

A myocardial scintigraphy showed a large antero-apical necrosis (30% of the anterior wall and 10% of the lateral wall were totally akinetic and non-viable). A coronarography identified a tri-truncal lesion, namely full occlusion of the second Cx segment and the second LAD segment, and tight stenosis of the first Mg, which was immediately stented. The patient was then scheduled for a CABG (LAD-Dg) and CD34^+^ cell injection which were performed on 22November, 2006. In total, 46.5 × 10^6^ CD34^+^ cells were trans-epicardially injected. Severe LV dysfunction occurred at the end of the CABG, requiring intra-aortic counter pulsion. On day14, the patient underwent an episode of global cardiac decompensation with oedema of the legs, which rapidly improved by increasing the dosage of diuretics. His LVEF was 20%. During the following weeks, things progressively improved, with partial recovery of his capacity for physical activities. The patient was discharged on 30 December, 2006.

At the 6-month assessment, the LV’s function had significantly improved, with an LVEF of 36%, a decrease in LVESV and LVEDV, and recovery of the kinetics and viability in the antero-apical segment and the infero-lateral junction. His quality of life was good enough (NYHA Class 2).

At the one-year FU, the patient was asymptomatic (NYHA Class1) and living normally, his LVEF was 53%, and his myocardial kinetics and viability were satisfactory, except in latero-median and latero-basal segments which remained hypokinetic and non-viable.

Throughout the 12 following years, the patient did very well, living normally at home, capable of walking relatively long distances (6–8 kms), and his LVEF being stable between 55 and 60%. However, from 2019, he began to present with increasing symptoms of Alzheimer’s disease, and he is now hospitalized in a specialized ward. He is 80 years-old.

### Patient 7

This patient was male, 58 years-old, a smoker, and overweight with hypercholesterolemia. On 15 November, 2006, the patient suffered the sudden onset of an intense and squeezing thoracic pain diffusing in the left dorsal and epigastric areas. He was transferred to the hospital 7 h after the appearance of the first symptoms, and the diagnosis of heart attack was made. His LVEF was 36% according to the echo 3D, which showed a large transmural anterior AMI with akinesia including the aneurismal apex, the apical third of the antero-septal and the infero-septal junctions, and the adjacent anterior wall. His LVESV and LVEDV were 90 mL and 146 mL respectively. The coronarography identified a bi-truncal lesion with a stenosis of the left coronary trunk of > 50%, an obstruction of the median LAD, a sub-obstructive stenosis of > 90% of the Cx ostium. A ^201^thallium scintigraphy additionally revealed a second infarcted area, that was non-transmural, affecting the infero-septo-median, basal, infero-median and basal areas, which were slightly hypokinetic but still viable. A PSE confirmed the non-viability of the whole transmural scar. The patient was dyspneic and presented with angina episodes. As his LVEF had decreased to 31% when his LVEDV was 154 mL and LVESV was 95 mL, a CABG plus CD34 + cell injection was proposed to the patient. In total, 55.1 × 10^6^ CD34 + cells were intra-epicardially delivered at the end of the CABG (LAD and Cx) on 10 January, 2007.

The general health of the patient improved rapidly, and at 1 month post-operation, he did not present any clinical symptom of HF with a slight increase in his LVEF (36%).

At the 6-month assessment, the patient was doing very well and his LVEF was 40%. The apex was dyskinetic and the antero-septal and infero-septal junction remained akinetic, but the adjacent inferior and anterior walls had recovered a good contractility and a good viability/perfusion.

After this time the patient began to resume sports and, at the 1-year FU, he went horseback riding every two days, cycling 10—15 kms or walking 6—8 kms in the interval. His LVEF was 42%, while the recovery of viability/reperfusion of the infero-lateral junction was obvious.

Throughout the following years, the patient remained very well, living normally, and his LVEF remaining stable at around 40–45%. In 2018 he underwent a successful surgery for an aneurism of the abdominal aorta. He is now 74 years-old, doing very well, still riding horses and cycling, at 16 years of FU.

## Discussion and Conclusions

In severe cases of AMI, such as those reported here, CABG surgery is only compassionate and does not prolong life expectancy, which was less than 5 years at the time of our study [7] Furthermore, the patients become more and more handicapped by the progressive decompensation of the CHF, the evolution of which is interspersed with numerous hospitalizations, which also leads to very high health care costs and makes it difficult to have any socio-professional life until death.

**Fig. 2:**
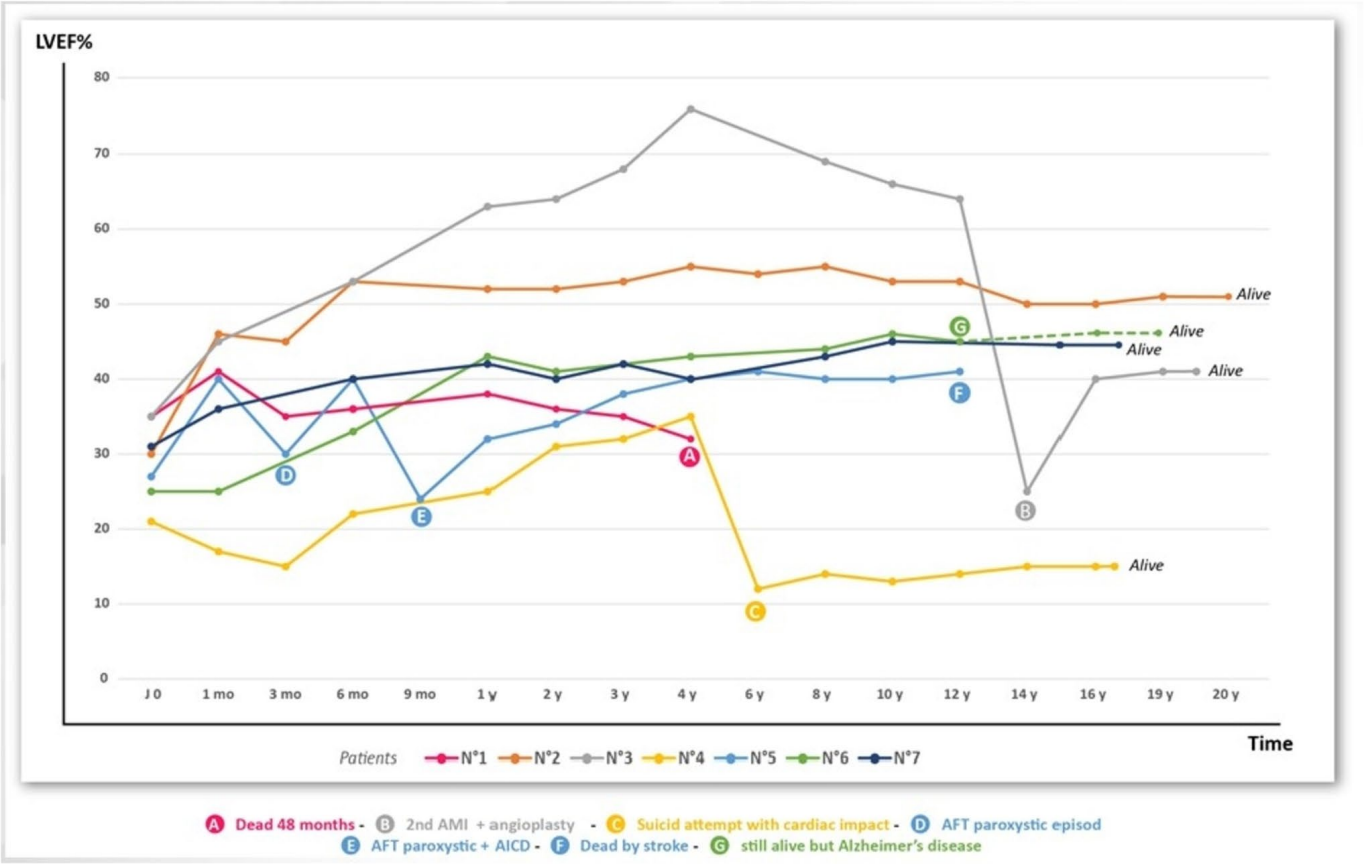
Patient’s long-term survey**.** Patients’ LVEF survey curves from D_0_ to 20 years. 6/7 patients were still alive 12 years after cells injection, one of them (patient 5) died by stroke at this time. The other five are still alive. AFT: atrial fibrillation tachycardia. AICD: automatic implantable cardioverter defibrillator

Thus, the long survival achieved by six out of seven of the AMI patients reported here (average: 17 years; range: 12–20 years)—three of whom having even avoided the heart transplantation they were initially recommended—raises questions when compared with the results of various statistical studies reporting short-term and long-term outcomes in AMI survivors in the era when contemporary pharmacotherapy of heart failure was not available. Particularly, the 10-year post-AMI mortality was 72.7% in a study covering 4 million patients within the same two decades as our patients’ survey period [3] compared with the rate of 14% achieved in our small cohort of patients (Fig. 2). Such a patients’ life expectancy enters the normal range, when compared with healthy controls. This long survival was associated with adequate living conditions allowing a normal socio-professional way of life. Most of these patients have never been re-hospitalized, except for the treatment of other diseases, and, apart from Patient 4 and more recently Patient 3—in whom a second AMI occurred 14 years after the cell therapy—they only took a few cardiac maintenance drugs daily, which altogether reduces the health costs by 50–70% compared with conventionally treated patients.

Interestingly, these clinical results correlate not only with structural repair and reperfusion of the damaged cardiac tissue, as shown by PSE, scintigraphy and angiography, but also with good enough recovery of myocardial contractility, as shown by echo 3D. As the ischemic area was never surgically reperfused, it is unlikely that the CABG operation contributed to the improvement in revascularization / reperfusion, or only contributes very little.

Various types and sources of cells have been experimented with for cardiac cell therapy within the past two decades. Intracardiac reinjection of autologous skeletal myoblasts did not achieved significant improvements in heart function, [8], which is not surprising as myoblasts are lineage-restricted progenitor cells that can only differentiate into skeletal muscle cells but not into cardiomyocytes. Overall, their clinical use was hindered by the frequent occurrence of severe ventricular arrhythmia related to differences in the electrical conduction between the injected myoblasts and the residual viable cardiomyocytes. Bone marrow (BM) mononuclear cells (MNCs) have been used in more than 60 randomized clinical trials enrolling up to 3000 AMI patients in total. Results of these trials were mainly neutral, or at best showed a modest improvement in LV function when more than 10^8^ MNCs were delivered [9]. In fact, BM-MNCs are not stem cells but a mix of various cells of hematopoietic lineages at different stages of maturation, and only contain a number of cells with stem cell-like features that is too low to efficiently replace the loss of cardiomyocytes destroyed by AMI. Mesenchymal stem cells (MSCs) are still rarer in the BM but can be easily expanded in vitro. They can also be isolated from the umbilical cord, Wharton’s jelly and adipose tissue. Their injection inhibits the formation of fibrosis in the border zone of the scar [10] and reduces its stiffness [11], thus limiting its extension [12]. Additionally, MSCs secrete soluble factors and exosomes that have proangiogenic activity allowing revascularization and reperfusion of the hibernated area surrounding the scar, but not of the scar itself [13]. Thus, improvements in cardiac function remain limited. The reality of the existence of endogenous cardiac stem cells (CSCs) was a dream that turned into a nightmare. Serious concerns about the integrity of the data published by the main group proclaiming their existence have led to the retraction, because of the charge of fraud, of more than 30 articles assessing the existence of CSCs. Further studies concluded that adult hearts contain no or only very few CSCs [14, 15]. What were called CSCs were more likely to be CD34^+^ cells deposited into the myocardium after AMI that were still capable of differentiating along the cardiac or endothelial pathways [6].

Indeed, several studies have shown that within hours of an infarction and for about a week, there is a 2 to threefold increase in the number of CD34^+^ cells circulating in the PB of patients [16, 17]. Experimental tests carried out in mice showed that these cells can attach to the periphery of the ischemic lesion to limit remodeling and thus the extension of the infarction. However, the number of cells involved is not sufficient to regenerate the damaged part of the heart. Human CD34^+^ cells injected directly into the heart of athymic rats that had undergone an experimental infarction have demonstrated their ability to differentiate in situ into cardiac and endothelial cells, resulting in anatomical and functional regeneration of the ischemic area [18, 19].

Different groups have also demonstrated the clinical interest in purified CD34^+^ cells in AMI [20–22], refractory angina [23, 24], or non-ischemic dilated cardiomyopathies [25]. However, the long-term outcomes of the patients enrolled in these trials were never recorded, except in the trial led by Vrtovec in which the patients were followed for 5 years, which is far short than the FU of our patients, which is the longest ever reported.

A significant improvement in heart function occurred from a threshold dose ≥ 10 × 10^6^ CD34^+^ cells in AMI [21, 22, 26]. It is likely that the more stem cells delivered, the better will be the clinical results, at least in AMI, given beating heart–related mechanical loss, cell escape, and in situ cell apoptosis and death [27–29]. Our patients were given 52 × 10^6^ CD34^+^ cells on average (range 29 × 10^6^ – 107 × 10^6^), much more than in other studies.

Cells injected directly into the damaged myocardium are probably retained more efficiently than cells infused via the coronary arteries, which are limited by their weak homing capacity to the injured area [30]. Several meta-analyses confirm a more significant clinical improvement of post-AMI heart function after trans-endocardial cell delivery compared to intra-coronary, several of them even concluding that the latter had no effect on clinical events or changes in LV function or remodeling [31, 32].

How might CD34^+^ cells restore the heart, if so, as they were considered for long to solely be hematopoietic stem cells (HSCs)? In fact, since the end of the 1990s, different groups have shown that the CD34 antigen was also borne by endothelial [33], liver, [34, 35], bone [36], and cardiac [6] progenitor cells. Additionally, we demonstrated that the G-CSF–mobilized CD34^+^ cells that were reinjected in our patients contained both small subpopulations of cells already engaged in the endothelial and cardiac pathways, and very immature cells that were still capable to differentiate into cardiac and endothelial progenitor cells when cultured in vitro for 14 days [6]. More recent works have shown that such immature CD34^+^ cells are actually pluripotent and they have been characterized as very small embryonic-like stem cells (VSELs) deposited during ontogenesis, [37]. They reside for life in the BM [38]. They are rare cells (0,01% of BM-mononuclear cells) but, interestingly, they can be significantly expanded in vitro while maintaining their pluripotency and competence to differentiate [39]. A contribution by injected purified VSELs to angiogenesis and cardiac repair has been shown in appropriate in vivo models [40].

As CD34^+^ cells contain both pre-committed cell subpopulations and VESLs, one can suggest that the first ones would begin the process of cardiac repair straight away, followed by stimulation of VSELs in a second phase, which would induce their long-term proliferation and differentiation. Together, these complementary actions would contribute to explain the progressiveness of clinical improvement over two years before reaching a stable plateau along the following years, as observed in our patients (6).

Finally, reviewing the literature and our present data, it seems that, to be potentially efficient in AMI, the injection of CD34^+^ cells should be carried out (1) at the highest cell dose possible, with a minimum threshold of 10 × 10^6^ CD34^+^ cells; (2) intramyocardially rather than intracoronary; and (3) before definitive remodeling of the scar, which induces the onset of CHF [41].

Of course, we are aware of the fact that our Pilot study was not randomized, not controlled, and only enrolled a small cohort of patients. However, its long-term outcomes were impressive enough to encourage us to launch a Phase II clinical trial (“EXCELLENT”, EUDRACT 2014–001476603), in which the AMI patients are randomized either to a cell-treated group receiving trans-endocardially autologous PB-CD34^+^cells, GMP expanded in vitro via a proprietary automated device and disposable cell culture kits we have developed [42, 43], or a standard-of-care group, to confirm these results. This trial is still ongoing but its preliminary data seem to be in line with those of our Pilot study.

## Data Availability

The original contributions presented in this study are included in the article; further inquiries can be directed to the corresponding author.
